# ﻿A new species of *Ranunculus* (Ranunculaceae) from Western Pamir-Alay, Uzbekistan

**DOI:** 10.3897/phytokeys.193.70757

**Published:** 2022-04-01

**Authors:** Natalia V. Shchegoleva, Elena V. Nikitina, Inom J. Juramurodov, Andrei A. Zverev, Orzimat T. Turginov, Anvarbek M. Jabborov, Ziyoviddin Yusupov, Davron B. Dekhkonov, Tao Deng, Hang Sun

**Affiliations:** 1 Department of Botany, Institute of Biology, Tomsk State University, 36 Lenin Ave., Tomsk 634050, Russia Tomsk State University Tomsk Russia; 2 Laboratory of Cadastre and Monitoring of Rare Plant Species, Institute of Botany of the Academy of Sciences of the Republic of Uzbekistan, 32 Durmon Yuli St., Tashkent, 100125, Uzbekistan Laboratory of Cadastre and Monitoring of Rare Plant Species, Institute of Botany of the Academy of Sciences of the Republic of Uzbekistan Tashkent Uzbekistan; 3 Laboratory Flora of Uzbekistan, Institute of Botany of the Academy of Sciences of the Republic of Uzbekistan, 32 Durmon Yuli St., Tashkent, 100125, Uzbekistan Laboratory Flora of Uzbekistan, Institute of Botany of the Academy of Sciences of the Republic of Uzbekistan Tashkent Uzbekistan; 4 University of Chinese Academy of Sciences, Beijing, China University of Chinese Academy of Sciences Beijing China; 5 Central Siberian Botanical Garden, Siberian Branch, Russian Academy of Sciences, 630090 Novosibirsk, Russia Siberian Branch, Russian Academy of Sciences Novosibirsk Russia; 6 Laboratory of Molecular Phylogeny and Biogeography, Institute of Botany of the Academy of Sciences of the Republic of Uzbekistan, 32 Durmon Yuli St., Tashkent, 100125, Uzbekistan Laboratory of Molecular Phylogeny and Biogeography, Institute of Botany of the Academy of Sciences of the Republic of Uzbekistan Tashkent Uzbekistan; 7 CAS Key Laboratory for Plant Diversity and Biogeography of East Asia, Kunming Institute of Botany, Chinese Academy of Sciences, Kunming 650201, Yunnan, China Kunming Institute of Botany, Chinese Academy of Sciences Kunming China

**Keywords:** Endemic, Hissar Range, Pamir-Alay, phylogenetic analysis, Ranunculales, *Ranunculastrum*

## Abstract

New data on the phylogeny of four rare and endemic species of RanunculusL.sect.Ranunculastrum DC. of western Pamir-Alai, one of which is new to science, have been obtained. *Ranunculustojibaevii* sp. nov., from the Baysuntau Mountains, Western Hissar Range of Uzbekistan, is described. The new species is closely related to *R.botschantzevii* Ovcz., *R.convexiusculus* Kovalevsk. and *R.alpigenus* Kom., but differs in the blade of the radical leaves, which is rounded-reniform, segments 3–5-dissected, each 2–5-partite with elongated, rounded apical lobes. A phylogenetic analysis, using both the nuclear ribosomal internal transcribed spacer (ITS) and *cp*DNA (*matK*, *rbcL*, *trnL-trnF*), was informative in placing *R.tojibaevii* in context with its most closely-related species. Discussion on the geographic distribution, updated identification key, a detailed description, insights about its habitat and illustrations are provided.

## ﻿Introduction

*Ranunculus* L., the largest genus in the Ranunculaceae Juss., includes ca. 600 genetically diverse species (Tamura 1995). The genus is distinguished by its high ecological-geographical diversity over a wide zonal spectrum ranging from the Arctic tundra through varied forests, steppes, deserts to exclusively aquatic habitats and high-altitude communities on nearly all continents (Paun et al. 2005). However, the main centres of speciation of *Ranunculus* are often in large mountain systems, where the formation of species is not only directly related to isolation, but also significantly depends on specific conditions of the highlands (Emadzade et al. 2015; Fernández Prieto et al. 2017; Shchegoleva 2018; Shchegoleva et al. 2020; Zverev et al. 2020).

More than 90 species of *Ranunculus* are distributed in Central Asia (Kovalevskaya 1972). Differentiation in the genus and the formation of locally endemic species are closely related to the history of the Tian Shan and Pamir-Alay Mountain formations. Here, more than half of the members of the genus are autochthonous representatives of the mountainous Central Asian flora, which arose in the process of regional adaptive diversification from ancient Mediterranean predecessors (Ovchinnikov 1971; Kamelin 1973). In the dry Central Asian seasonal climate, the features of these species are observed only in the short-term hydrothermal period of active vegetative growth.

*Ranunculustojibaevii* was first discovered in 2013 on the Baysuntau Highlands (Khodzha-Gurgur-ata Mountain) on the south-western spur of the Hissar Range (Pamir-Alay). The populations were detected again in 2019 while working on the *Flora of Uzbekistan Project* (Sennikov et al. 2016). It should be noted that the flora of the Western Pamir-Alay is characterised by a high taxonomic diversity (Kamelin 1973; Vasilchenko and Vasileva 1985; Tojibaev et al. 2016; Makhmudjanov et al. 2019; Yusupov et al. 2020).

The morphological features indicated that the unknown plants belonged to R.subg.Ranunculussect.Ranunculastrum (Hörandl and Emadzade 2012; Baltisberger and Hörandl 2016). The main differences between sect. Ranunculastrum and other sections of *Ranunculus* are the presence of a triangular beak equal to or longer than the achene body, a receptacle completely glabrous, a taproot partly tuberous and a mostly elongate fruit (Hörandl and Emadzade 2012).

The unknown plants closely resembled *R.botschantzevii* Ovcz. (Ovchinnikov 1941) and *R.convexiusculus* Kovalevsk. (Kovalevskaya 1972), as well as *R.alpigenus* Kom. (Komarov 1896) in their main morphological features. The molecular results presented here also clearly substantiated its independent taxonomic status. In this study, we present a morphological description of these plants, which we named *Ranunculustojibaevii* Schegol. & Turginov. Figures showing its features, a map of its distribution, taxonomy and an identification key separating it from the most closely-related species are also provided.

## ﻿Materials and methods

Morphological observations and measurements of *R.tojibaevii* were made on two populations; in total, 34 individuals were compared. Voucher specimens have been deposited in the National Herbarium of Uzbekistan – TASH (Tashkent, Uzbekistan). Additionally, two closely-related species, *R.convexiusculus* and *R.botschantzevii*, from the same territory and high-altitude regions were examined. Herbarium specimens at TASH, TAD, LE, FRU, AA, MW, LE and TK were also compared. Studies of closely related species were performed during field expeditions to Uzbekistan, Tajikistan and Kyrgyzstan, from 2017–2021 (Shchegoleva et al. 2020). The distribution map was generated in ESRI ArcGIS 10 software using GPS coordinates (www.esri.com). The conservation status was established, based on IUCN Criteria (IUCN 2019).

### ﻿Molecular methods

#### DNA extraction, amplification and sequencing

DNA isolation was performed using a Plant Genomic DNA Kit (TIENGEN Biotech, Beijing, China) according to the manufacturer’s protocol. CTAB extraction protocol with some modifications was used to extract genomic DNA from herbarium specimens of *R.alpigenus* (Doyle and Doyle 1987).

Selected nuclear DNA regions ITS1-ITS2 (for herbarium specimen *R.alpigenus*) and ITS1-ITS4; plastid DNA regions *matK*, *rbcL* and *trnL-F* were amplified on a thermal cycler (BioRad) using the 2X PCR Taq Plus MasterMix with dye (Applied Biological Materials Inc., Canada). Amplification of the DNA regions was carried out by using primers of the forward and reverse primer sets (TsingKe, China) (Table 1).

**Table 1.** Primers used in this study.

Primer name	Sequences (forward / reverse)	DNA fragment size, bp	Primer source
ITS1-18S ITS4-26S	5’-TCCGTAGGTGAACCTGCGG-3’ 5’-TCCTCCGCTTATTGATATGC-3’	~ 700 bp	White et al. (1990)
ITS1	5’-TCCGTAGGTGAACCTGCGG -3’	~ 650 bp	White et al. (1990)
ITS2	5’-GCTGCGTTCTTCATCGATGC-3’		
*matK-390F matK-1326R*	5’-CGATCTATTCATTCAATATTTC-3’ 5’-TCTAGCACACGAAAGTCGAAGT-3’	~ 900 bp	Cuenoud et. al. (2002)
*trnL-F_F trnL-F_R*	5’-CGAAATCGGTAGACGCTACG -3’ 5’-ATTTGAACTGGTGACACGAG-3’	~ 900 bp	Taberlet et al. (1991)
*rbcLaF rbcLaR*	5’-ATGTCACCACAAACAGAGACTAAAGC-3’ 5’-GTAAAATCAAGTCCACCRCG-3’	~ 600 bp	Kress and Erickson (2007)

To obtain sequences of the genes of interest, PCR amplification was carried out according to the following parameters (except *R.alpigenus*): for ITS1-ITS4, initial denaturation for 3 min at 94 °C, followed by 35 amplification cycles: 30 s at 94 °C, 30 s at 50–54 °C, 1 min at 72 °C; elongation 7 min at 72 °C; for *matK*– an initial denaturation for 3 min 94 °C, followed by 35 amplification cycles: 30 s 94 °C, 1 min 51 °C, 1 min 72 °C; final extension 10 min at 72 °C; for *rbcL*, an initial denaturation for 4 min 95 °C, followed by 34 amplification cycles: 1 min 94 °C, 1 min 50 °C, 1 min 72 °C; final extension 10 min at 72 °C; for *trnL-F*, an initial denaturation for 3 min 94 °C, followed by 32 amplification cycles: 45 s 94 °C, 45 s 50 °C, 1 min 72 °C; final extension 8 min at 72 °C.

PCR amplification for *R.alpigenus* was performed for ITS1-ITS2, with the following programme: initial denaturation at 94 °C/5 min; 35 amplification cycles at 94 °C/30 s, at 54 °C/30 s, at 72 °C/ 45 s; elongation at 72 °C/7 min; for *rbcL*, 94 °C/4 min, 34 cycles: 94 °C/30 s, 54 °C/ 45 s, 72 °C/45 s; final extension at 72 °C/10 min.

#### Taxon sampling

To determine the taxonomic status and systematic position of *R.tojibaevii*, we sampled 24 species of *Ranunculus*. New nDNA (ITS) and *cp*DNA intergenic spacers (*matK*, *rbcL*, *trnL-trnF*) sequences for nine species were generated. We also used available sequences of 15 *Ranunculus* species from GenBank (www.ncbi.nlm.nih.gov/Genbank) (Table 2). We used RanunculussubgenusAuricomus as the outgroup (Hörandl and Emadzade 2012; Almerekova et al. 2020).

**Table 2.** Accession numbers of samples used for phylogenetic analyses of *Ranunculus* (* newly-generated sequences).

Species	GenBank accession number			
	ITS	*matK*	*rbcL*	*trnL-F*
*Ranunculusacris*	AY680167	AY954199	HQ590232	–
R.alpigenus*	OM283824	–	OM287560	–
*R.arvensis*	HQ650550	HQ650551	MK925091	–
*R.auricomus*	FM242803	FM242739	JN893758	–
R.botschantzevii*	MW540744	MW748677	MW748685	MW748693
R.convexiusculus*	MW540743	MW748676	MW748684	MW748692
*R.flammula*	AY680185	AY954204	MK526480	–
*R.glaberrimus*	KP687273	JF509974	MG247649	–
*R.inamoenus*	KP687279	KP687302	MG249011	–
*R.japonicus*	EU591982	AY954200	MH657741	DQ410744
R.leptorrhynchus*	MW737444	MW748673	MW748681	MW748689
R.linearilobus*	MW737445	MW748674	MW748682	MW748690
*R.lingua*	AY680184	AY954206	JN892742	–
*R.muricatus*	DQ410718	AY954191	HM850296	DQ410740
R.paucidentatus*	MW540747	MW748679	MW748687	MW748695
*R.pygmaeus*	KP687287	KP687310	KC483860	–
R.regelianus*	MW737446	MW748675	MW748683	MW748691
*R.repens*	MT271835	HM565166	MK925397	EU382995
*R.sceleratus*	MT271836	GU257993	AB517148	DQ410746
*R.sulphureus*	JF509969	JF509983	KC483870	–
R.talassicus*	MW540748	MW748680	MW748688	MW748696
R.tojibaevii*	MW540745	MW748678	MW748686	MW748694
R.tojibaevii*	OM278385	OM287558	OM287559	–
*R.trichophyllus*	KC620483	AY954133	L08766	–
*R.turneri*	FM242817	FM242741	MG249550	–

#### Phylogenetic analyses

Sequence alignments were performed using ClustalW (Thompson et al. 2002) as implemented in MEGA X software (Kumar et al. 2018). The best partitioning scheme for the combined dataset contained two partitions: the ITS data; and the three plastid sequences data (*matK*, *rbcL*, *trnL-trnF*). Phylogenetic reconstruction was first conducted separately, based on the nuclear and the plastid data. Visual inspection determined that differences between the nuclear and the plastid trees were solely due to resolved/collapsed clades. No topological incongruence with a high support value (posterior probabilities and bootstrap percentages) was found. To further test whether the nuclear and plastid data could be combined for phylogenetic reconstruction, the incongruence length difference (ILD, Farris et al. 1995) test was conducted in PAUP* 4.0a169 (current) by using only the informative sites, heuristic search, tree-bisection-reconnection (TBR) branch-swapping algorithm, simple addition sequence and 1,000 replicates. The ILD test between the nuclear and the plastid data found *p* = 0.322, indicating insignificant support for incongruence between the two datasets. Therefore, the nuclear and the plastid sequences were combined into one dataset for phylogenetic analyses using SequenceMatrix software (Vaidya et al. 2011).

Phylogenetic trees were reconstructed using Maximum Likelihood (ML), Maximum Parsimony (MP) and Bayesian Inference (BI). For ML, we employed raxmlGUI 2.0 (Edler et al. 2020), with 1,000 bootstrap replicates and, for BI, we used MrBayes v.3.1.2 (Huelsenbeck and Ronquist 2001) with 10,000,000 generations with random trees sampled every 1,000 generations. In the latter analysis, after discarding the first 25% trees as burn-in, a 50% majority-rule consensus tree was constructed from the remaining trees to estimate Posterior Probabilities (PP). For analyses, a model of nucleotide substitution was selected, based on the Akaike Information Criterion (AIC) using jModelTest2 on XSEDE (www.phylo.org). Phylogenetic analyses were also performed with the MP method using PAUP* 4.0a169. The MP bootstrap analysis was performed with heuristic search, TBR branch-swapping, 1,000 bootstrap replicates, random addition sequence with ten replicates, a maximum of 1,000 trees saved per round. Trees were visualised in FigTree v.1.4.0 (Rambaut 2012).

## ﻿Results and discussion

**Figure 1.**
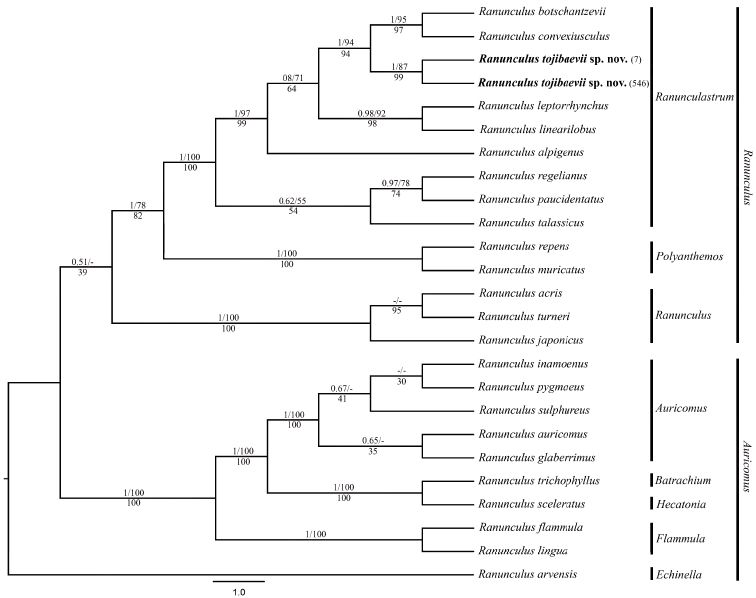
Bayesian tree based on combined nuclear (ITS) and plastid (*matK*, *rbcL*, *trnL-trnF*) sequence data showing phylogenetic position of *Ranunculustojibaevii* Schegol. & Turginov in R.sect.Ranunculastrum. Bayesian Posterior Probability (PP) / Maximum Parsimony (MP) is given on each branch, respectively; Maximum Likelihood (ML) is below branches. The classification is according to Hörandl and Emadzade (2012). * denotes the *Ranunculus* species analysed in this study. The new species is highlighted in bold.

The phylogenetic tree, based on the nuclear and plastid sequences (Fig. 1), showed that *R.tojibaevii* is sister to *R.convexiusculus* and *R.botschantzevii* with high support values PP = 1, MP = 94% and ML = 94%. *Ranunculustojibaevii*, *R.convexiusculus* and *R.botschantzevii* formed a clade with well supported values (PP = 0.8, MP = 71% and ML = 64%).

The results of the phylogenetic analysis were similar to the results of Hörandl and Emadzade (2012) and Almerekova et al. (2020). Species of R.sect.Ranunculastrum are of particular interest. In our case, these native Asian species are mainly limited to the foothills and mountains of Central Asia (southern Kazakhstan, Uzbekistan, Kyrgyzstan, Tajikistan and Turkmenistan), as well as to the mountains of Afghanistan, Iran and Pakistan.

It is obvious that R.sect.Ranunculastrum in Central Asia is heterogeneous. The species forming sub-clusters in this section differ in their area of distribution, altitude confinement and time of origin, thereby confirming the neoendemic nature and origin of *R.tojibaevii*.

## ﻿Taxonomy

### ﻿ Ranunculus tojibaevii

Taxon classificationPlantaeRanunculalesRanunculaceae

Schegol. & Turginov sp. nov.

200BBEC3-EDDC-5391-B0F9-79F409C8D487

urn:lsid:ipni.org:names:77296907-1

Figs 2 , 3

#### Diagnosis.

Similar to *R.botschantzevii*, *R.convexiusculus* and *R.alpigenus* morphologically, but differing in the rounded-reniform radical leaves dissected into 3–5 segments, each 2–5-partite into elongated lobes rounded at the apex (Fig. 4). *Ranunculustojibaevii* differs from *R.alpigenus* in having fewer levels of leaf blade dissection. It differs from *R.convexiusculus* in the dissection of the radical leaf blades, larger flowers and having somewhat white, bristle-like hairs on the root collar and also from *R.botschantzevii* by the rounded apical lobes of the basal leaves and more xeromorphic habit.

**Figure 2.**
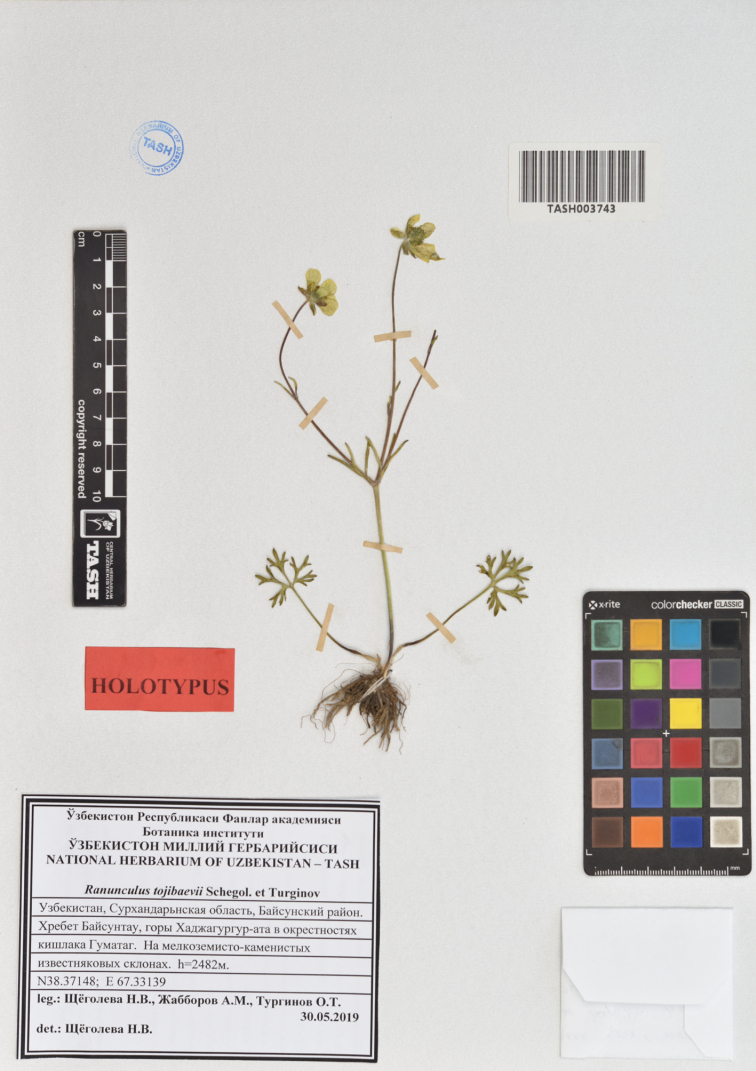
*Ranunculustojibaevii* Schegol. & Turginov (*Shchegoleva*, *Jabborov*, *Turginov*, holotype TASH-003743).

#### Type.

Uzbekistan. Hissar Ridge, Baysuntau, Khodzha-Gurgur-ata Mountains, vicinity of the Village Gumatag, 38°22.2888'N, 67°21.0834'E, 2482 m a.s.l., 30 May 2019, N. Shchegoleva, A. Jabborov, O. Turginov (holotype: TASH-003743; isotypes: TASH-003748, TASH-003749, TASH-003750, TK-002339).

**Figure 3.**
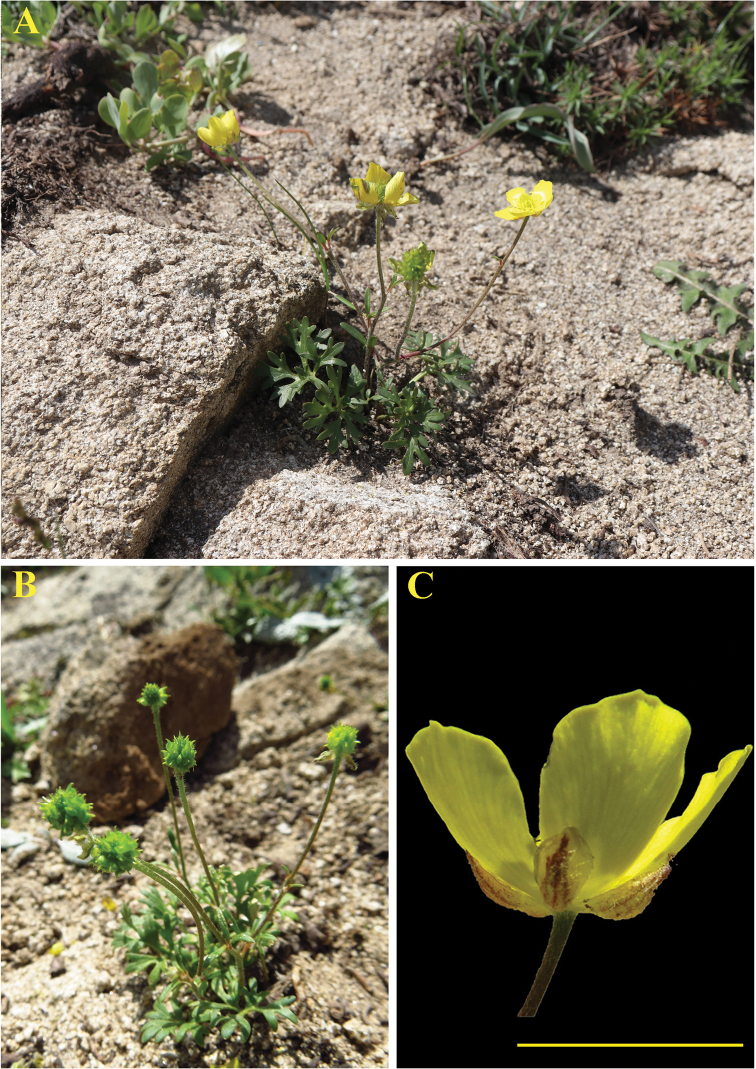
*Ranunculustojibaevii* Schegol. & Turginov. Habitat (**A** flowering **B** fruiting) and flower (**C**). Scale bar: 1 cm.

#### Description.

*Herbs* perennial. *Roots* dimorphic, some roots subulate, up to 0.5 mm thick, storage roots palmately-thickened, ca. 2.5 mm thick; root collar with milk-white bristle-like hairs. *Stems* 10–12(–15) cm tall, up to 2 mm diam., erect, branched, pubescent with white curly hairs, 1–3-flowered. *Leaves* dimorphic, radical leaves 2–3, 1.6–2 × 1.5–2.2 cm, blade rounded-reniform, 3–5-dissected, segments 2–5-partite, elongate, lobes apically rounded; cauline leaves 1–2, petiole short, slender, blade trisected, lobes 0.6–0.9 × 0.1–0.2 cm, oblong-lanceolate. *Flowers* 1.6–2.4 cm diam., sepals 0.4–0.6 mm long, ovate-concave, sparsely white pubescent; petals 0.9–1.2 cm long, well-developed, ovate, apex rounded. Infructescence globose-ovoid; receptacle oblongoid, glabrous; achenes 1–1.8 mm long, with white bristle-like hairs; beak hamate-curved.

**Figure 4.**
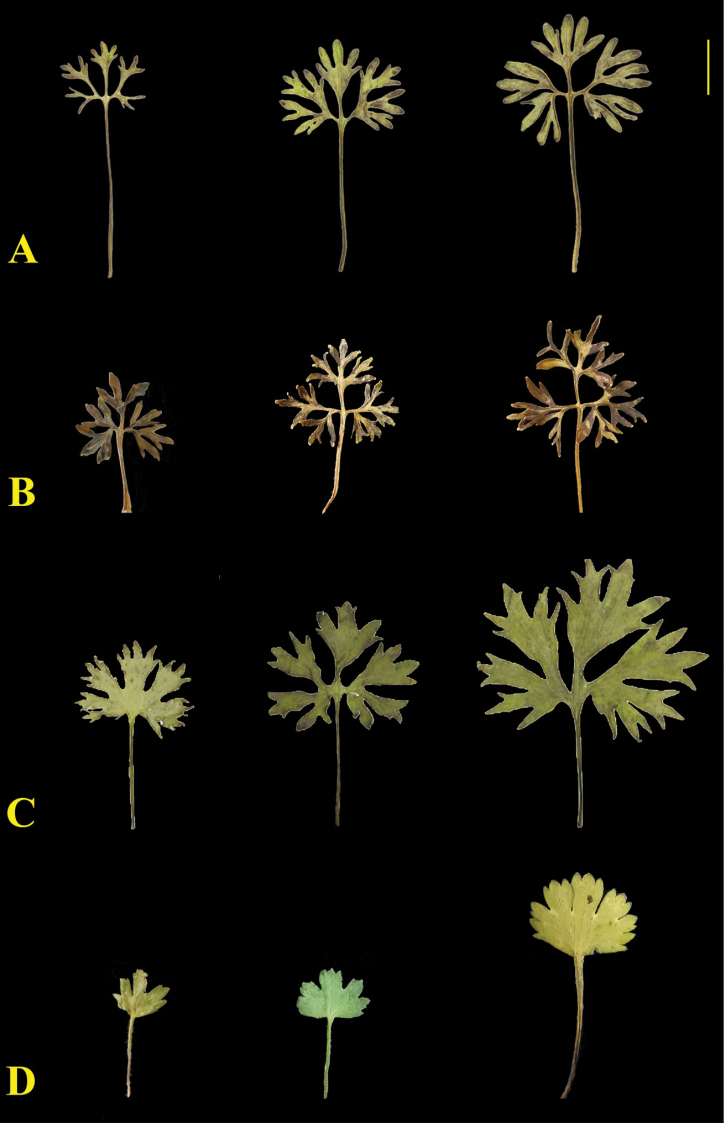
Series of basal leaves in related species **A***Ranunculustojibaevii* (from the holotype) **B***R.alpigenus***C***R.botschantzevii***D***R.convexiusculus*. Scale bar: 1 cm.

#### Specimen seen (paratype).

Uzbekistan. Pamir-Alay, South-western spurs of the Hissar Ridge, Baysuntau, vicinity of the Village Gumatag, amongst the stones, 4 June 2013, O. Turginov (TASH-003754).

#### Phenology.

Flowering in May. Fruiting in May and June.

#### Distribution.

**Figure 5.**
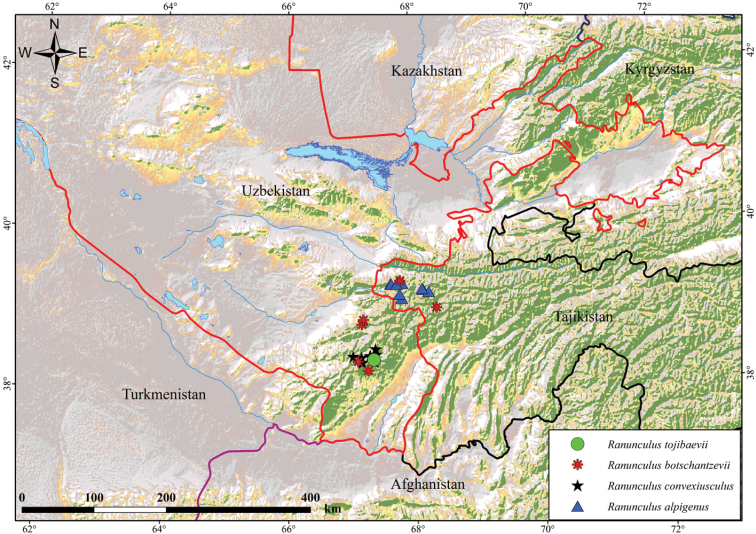
Distribution of *Ranunculustojibaevii*, *R.alpigenus*, *R.botschantzevii* and *R.convexiusculus*.

*Ranunculustojibaevii* is distributed in the Khodzha-Gurgur-ata of the Baysuntau Mountains area of Hissar Ridge (Fig. 5). The closely-related *R.convexiusculus* is endemic to Central Asia and *R.botschantzevii* is endemic to the Western Pamir-Alay.

#### Habitat and plant associations.

*Ranunculustojibaevii* is rupicolous on southern and western exposed limestone outcrops and in cracks and crevices of large boulders at 2,450–2,500 m. a.s.l. The region is alpine and rather xerophytic. The common taxonomic composition of phytocenosеs includes *Cerasusamygdaliflora* Nevski (Rosaceae), *Corydalisledebouriana* Kar. & Kir. (Papaveraceae), *Cousiniaregelii* C.Winkl. (Asteraceae), *Eremurusregelii* Vved. (Asphodelaceae), *Gageagymnopoda* Vved. (Liliaceae), *Iriskhassanovii* Tojibaev & Turginov, *I.parvula* (Vved.) T.Hall & Seisums, *I.stolonifera* Maxim. (all Iridaceae), *Rheummaximowiczii* Losinsk. (Polygonaceae), *Tulipalanata* Regel (Liliaceae) and *Ziziphorapamiroalaica* Juz. (Lamiaceae).

#### Conservation status.

*Ranunculustojibaevii* is a local, narrowly distributed endemic, represented by two or three individuals per m^2^ within an area of < 500 m^2^. It should therefore be assigned the status EN (Endangered), Criteria B1 ab(i, ii, iii)+ B2 ab(i, ii, iii), following the IUCN Standards and Petitions Committee (IUCN 2019).

#### Notes.

*Ranunculustojibaevii* differs from closely-related species by its habitat on well-heated limestone outcrops, as well as in cracks and crevices of large boulders at ca. 2,500 m a.s.l., which is atypical of related species. *Ranunculusconvexiusculus* is on clayey-stony soil, less often on slopes of fine earth, at 2,000–2,600 m a.s.l. *Ranunculusbotschantzevii* is hygrophilous in wet mountain meadows with melting snow and on slopes of fine clayey soil at 2,400–3,500 m a.s.l. *Ranunculusalpigenus* grows on slopes of fine soil of the alpine belt at 2,800–4,000 m a.s.l. All these species are endemic to the western Pamir-Alay. The vicariant species to *R.alpigenus* is *R.badachschanicus* Ovcz. & Koch. from the western Pamirs.

#### Etymology.

*Ranunculustojibaevii* is named after Komiljon Tojibaev, a leading botanist, professor and academician from Uzbekistan who actively promotes the botanical sciences in Central Asia.

**Table 3.** Comparison of *R.tojibaevii*, *R.botschantzevii*, *R.convexiusculus* and *R.alpigenus.*

	*R.tojibaevii*	*R.botschantzevii*	*R.convexiusculus*	*R.alpigenus*
**Blade of radical leaves**	rounded-ovate, 3–5-dissected, segments further 2–5-dissected, lobules elongated, rounded at apex	triangular-reniform, 3–5-partite, segments narrowly cuneate, unequally and subacutely dentate	reniform, dissected 1/3 to nearly 1/2 of its length, lobes broad incised-dentate	broadly ovate, dissected into pinnatipartite segments, segments tripartite, terminal lobules oblong
**Sepals**	narrowly elliptic, concave, less than half as long as petals, with long reclinate hairs	elliptic, concave, some shorter than the petals, with sparse, long reclinate hairs	elliptic, concave, half as long as petals, with short reclinate spreading hairs	elliptic, concave, with scattered hairs
**Petals**	oblong-obovate, greenish-yellow, base cuneate, margin undulate	obovate, bright yellow, becoming dark when dry, base broadly cuneate, margin undulate	very broadly ovate, golden yellow, base cuneate, margin slightly undulate	oblong-ovate, yellow-green, base narrowly cuneate, marginundulate
**Achenes**	1.0–1.8 mm long, asymmetrically ovate, slightly convex, with semi-appressed hairs	2.2–2.5 mm long, oblong, slightly laterally compressed, with appressed hairs	1.8–2.5 mm long, oblong, slightly convex, with appressed hairs	1.5–2.0 mm long, asymmetrically obovate, laterally compressed, with scattered not appressed hairs

### ﻿Key to *Ranunculustojibaevii* and similar species (Table 3)

**Table:** 

1	Blades of basal leaves broadly ovate, dissected into pinnatipartite segments, with tripartite-oblong terminal lobules	***R.alpigenus***
–	Radical leaves 3–5-dissected or lobed-incised	**2**
2	Blade of radical leaves 1/3 or nearly 1/2 unequally partite into broad incised-dentate lobes	***R.convexiusculus***
–	Blade of radical leaves 3–5-dissected	**3**
3	Blade of basal leaves triangular-reniform, 3–5-dissected almost to the base, wedge-shaped segments, unequally sharp-toothed	***R.botschantzevii***
–	Blade of radical leaves is round-reniform, 3–5-dissected, each section divided into 2–5 elongated lobules; apex of lobules rounded	***R.tojibaevii***

## Supplementary Material

XML Treatment for Ranunculus tojibaevii
